# Diffusion of Lipid Nanovesicles Bound to a Lipid Membrane Is Associated with the Partial-Slip Boundary Condition

**DOI:** 10.1021/acs.nanolett.1c02092

**Published:** 2021-08-17

**Authors:** Erik Olsén, Silver Jõemetsa, Adrián González, Paul Joyce, Vladimir P. Zhdanov, Daniel Midtvedt, Fredrik Höök

**Affiliations:** † Department of Physics, Chalmers University of Technology, SE-41296 Göteborg, Sweden; ‡ UniSA: Clinical and Health Sciences, University of South Australia, 5000 Adelaide, Australia; § Boreskov Institute of Catalysis, Russian Academy of Sciences, Novosibirsk 630090, Russia; ∥ Department of Physics, University of Gothenburg, SE-41296 Göteborg, Sweden

**Keywords:** Multivalent interactions, single-particle tracking, lipid vesicles, confined diffusion, slip length

## Abstract

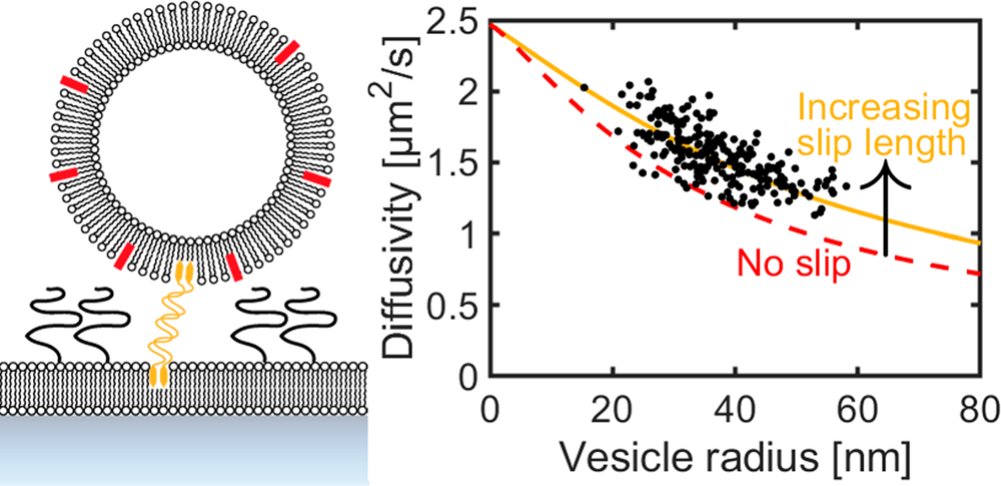

During diffusion of nanoparticles bound to a cellular membrane by ligand–receptor pairs, the distance to the laterally mobile interface is sufficiently short for their motion to depend not only on the membrane-mediated diffusivity of the tethers but also in a not yet fully understood manner on nanoparticle size and interfacial hydrodynamics. By quantifying diffusivity, velocity, and size of individual membrane-bound liposomes subjected to a hydrodynamic shear flow, we have successfully separated the diffusivity contributions from particle size and number of tethers. The obtained diffusion-size relations for synthetic and extracellular lipid vesicles are not well-described by the conventional no-slip boundary condition, suggesting partial slip as well as a significant diffusivity dependence on the distance to the lipid bilayer. These insights, extending the understanding of diffusion of biological nanoparticles at lipid bilayers, are of relevance for processes such as cellular uptake of viruses and lipid nanoparticles or labeling of cell-membrane-residing molecules.

Many biological processes involve interfacial biomolecular interactions in confined geometries. In the case of nanoparticles near biological interfaces, nanoparticle diffusivity can be used to estimate both the nature of the interfacial interactions and nanoparticle size.^1−3^ However, confined nanoparticle diffusion is significantly influenced by hydrodynamical boundary conditions in general and especially when the distance between the nanoparticle and an interface is shorter than the size of the particle,^1,4^ which naturally occurs during the initial interaction between biological nanoparticles and cellular membranes.^5^ Characterizing nanoparticle diffusivity at such a short distance to an interface is therefore crucial for in-depth understanding of fundamental hydrodynamical effects, which are relevant in a multitude of biological processes, including viral infection,^6^ exosome-controlled intracellular communication,^7^ nanoparticle-assisted drug delivery,^8^ as well as when nanoparticles are used as labels for molecules residing in laterally fluid cell membranes.^9^

Since the hydrodynamics around hydrophilic interfaces often is well-described by the no-slip boundary condition,^10^ this boundary condition is typically employed for biological interfaces, as they often consist of lipid bilayers with hydrophilic headgroups facing the surrounding fluid. Previous studies using a dynamic surface force apparatus to determine the boundary conditions of supported lipid bilayers (SLBs) indicate that the gel-phase DPPC (dipalmitoylphosphatidylcholine) bilayers appear to fulfill this condition,^11,12^ whereas a slip length of 6 ± 0.5 nm has been observed for fluid DOPC (dioleoylphosphatidylcholine) bilayers.^11^ Hence, the assumption of no slip is not necessarily generally valid and should in many situations be replaced by partial slip, characterized by a slip length *b* defined as the distance below the surface at which an extrapolation of the velocity profile parallel to the interface becomes zero (see schematic illustration in Figure 1a).^1,13,14^ However, it remains difficult with existing methods^15−17^ to directly measure slip for nanoparticles in general and biological nanoparticles in particular, primarily due to sample heterogeneity, small buoyancy forces, and a lack of means to simultaneously determine both nanoparticle size and mobility. Thus, there is a need for new approaches to quantify the hydrodynamic boundary conditions for nanoparticle systems.

**Figure 1:**
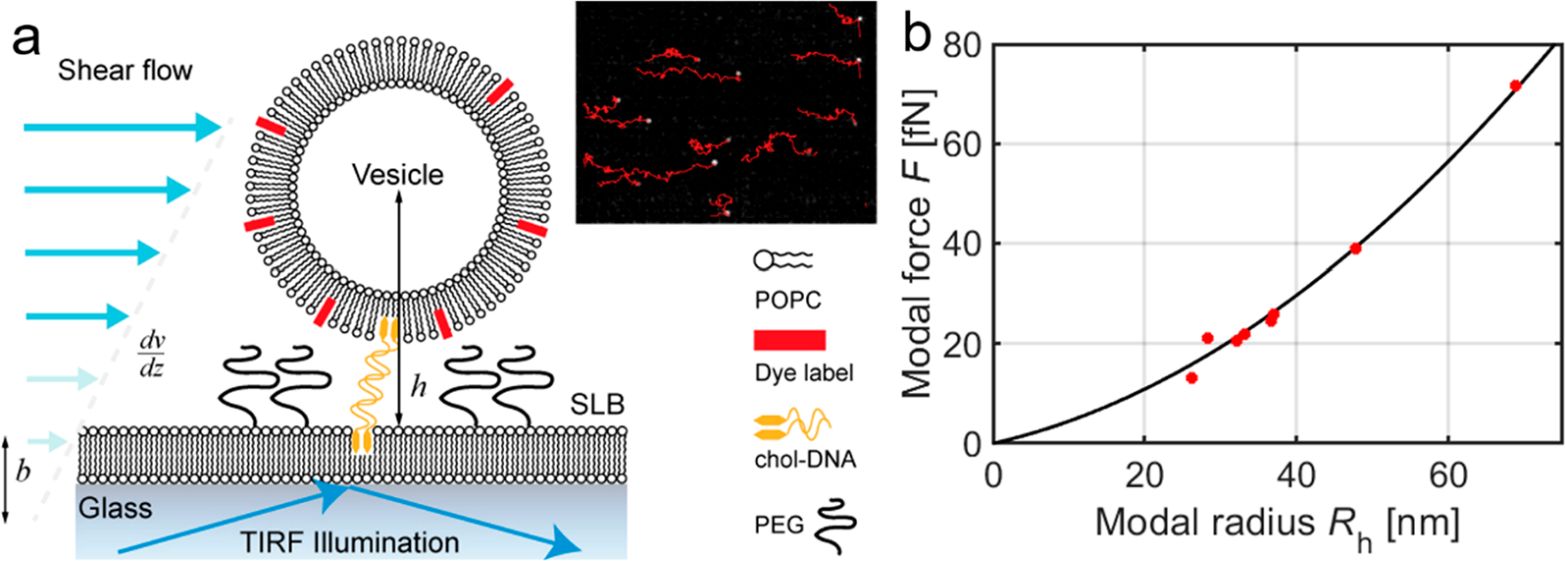
Illustration of the two-dimensional flow nanometry (2DFN) concept. (a) Nanoparticles are linked to a supported lipid bilayer (here using cholesterol–DNA tethering) within a microfluidic channel. A shear flow is applied, and the movement of the vesicles is tracked using total internal reflection fluorescence (TIRF) imaging (see inset). Here, *b* is the slip length of the lipid bilayer, and *h* is the distance to the surface from the center of the vesicle. (b) The calibration parameters in eq 1 are obtained by relating the modal hydrodynamic radius measured in bulk using NTA to the modal force measured using 2DFN.

In the context of nanoparticles near fluid lipid bilayers, the distance between a nanoparticle and an SLB is often constrained due to the formation of ligand–receptor pairs or, in other words, molecular tethers,^18^ making it comparable to the length scale of the potential slip. Since the motion of tethered nanoparticles is largely determined by the diffusivity of the linker,^19^ it is common to neglect the potential influence on mobility of the nanoparticle itself.^9^ It was recently shown, however, that in the limit of single or few tethers between gold nanoparticles and an SLB, the nanoparticle size had an appreciable influence on the resulting mobility down to particle diameters as small as 10 to 20 nm.^20^ This suggests in turn that quantification of the size-dependent mobility of nanoparticles bound to an SLB could offer a novel opportunity to gain new insights about the hydrodynamic boundary conditions and confined nanoparticle diffusion near planar interfaces of both experimental and theoretical importance, especially since the current theoretical representations of confined diffusion contain several approximations that are in need of experimental validation. However, in contrast to synthetic nanoparticles with narrow size distributions, quantification of diffusion-size relations for biological nanoparticles, which typically have broad size distributions, requires means to simultaneously determine both size and mobility on the level of individual nanoparticles.

Herein, we take a new step toward addressing the challenge of measuring the hydrodynamic boundary conditions for biological nanoparticles using two-dimensional flow nanometry (2DFN), which enables simultaneous quantification of both size and mobility for synthetic lipid vesicles and cell-derived extracellular vesicles (EVs) when bound to an SLB.^21^ 2DFN is based on optical tracking of the flow-induced motion of nanoparticles tethered to an SLB formed on the floor of a microfluidic channel (Figure 1a), which enables simultaneous measurement of the flow-induced velocity, *v*_*x*_, and the mobility of the combined nanoparticle–tether system, μ = *Dk*_B_*T* (*D* is here the measured diffusion constant). When combining these independently determined parameters, they can be used to calculate the hydrodynamic force, *F* = *v*_*x*_μ^–1^, acting on the particle.^21,22^ Assuming spherical nanoparticles, which is reasonable considering the deformation force for a ∼50 nm radius lipid vesicle (Supporting Information, Section 2.2), *F* can be related to the 2DFN nanoparticle radius, *R*_FN_, as^21^
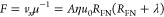
1where η is the dynamic viscosity, *u*_0_ is bulk liquid flow velocity, and *A* and λ are channel- and interface-specific calibration parameters.^21^ Here, the calibration was performed by associating the maximum of the distribution of hydrodynamic radius, *R*_h_, obtained from nanoparticle tracking analysis (NTA) of suspended nanoparticles, with the maximum of the measured *v*_*x*_μ^–1^ distribution as shown in Figure 1b (Supporting Information, Section 2.3). Since the measured size distribution of particles at the surface is not necessarily identical to the size distribution in bulk, *R*_FN_ is close, but not strictly identical, to *R*_h_. In fact, the corresponding difference is small, ∼1 nm for ∼30 nm radius vesicles (Supporting Information, Section 2.4), and accordingly, we set *R*_FN_ = *R*_h_.

In general, the mobility of the combined nanoparticle–tether system depends on both the nanoparticle mobility μ_NP_ and the mobility of an individual tether μ_T_.^23^ To specifically quantify the mobility of the nanoparticle, it is necessary to disentangle the two mobility contributions, keeping in mind that the nanoparticle may be bound to the SLB by more than one tether. Concerning these aspects, we note that the nanoparticle and tether mobilities are determined by two approximately independent frictions: between the nanoparticle and solution and between tethers and lipids, respectively. According to previous experiments,^19,20^ the friction associated with μ_T_ agrees well with the free-draining model^24^ and can thus be represented as a sum of independent frictions from the individual tethers. By also considering that the frictions are inversely proportional to mobilities, we have
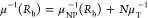
2where *N* is the number of tethers. Since *N* can here only attain discrete values, this suggests that the two different mobility contributions in eq 2 can be separated, provided that particle populations linked by different numbers of tethers can be distinguished from the combined particle size and mobility measurements offered by 2DFN.

To explore this opportunity, 2DFN was employed to analyze fluorescently labeled POPC (1-palmitoyl-2-oleoyl-*sn*-glycero-3-phosphocholine) lipid vesicles, with *R*_h_ centered around 35 nm, as well as EVs, with *R*_h_ centered around 45 nm, tethered to an SLB (consisting of POPC and 0.5 mol% PEG2000-PE (1,2-dioleoyl-*sn*-glycero-3-phosphoethanolamine-*N*-[methoxy(polyethylene glycol)-2000])) formed on the glass floor of a rectangular PDMS microfluidic channel (height 80 μm, width 400 μm). The POPC vesicles were prepared by the freeze–thaw extrusion method (Supporting Information, Section 1). Experimental details for the EV data are described in ref (25). The vesicles were subjected to a volumetric flow of TE buffer (50–200 mM NaCl, 10 mM TRIS, 1 mM Na_2_EDTA) at 30 μL/min. The POPC vesicles contained 2 mol% ATTO488PE, whereas the EVs were labeled using the self-inserting lipophilic dye 3,3-dioctadecyl-5,5-di(4-sulfophenyl)oxacarbocyanine (SP-DiO). The particles were linked to the SLB using complementary DNA–cholesterol tethers (Supporting Information, Section 1.3), with a total length of about 15 nm, and imaged using total internal reflection fluorescence (TIRF) microscopy.

To test whether the two experimentally obtained mobility-dependent particle properties, *v*_*x*_ and *D*, can be used to resolve the expected discrete effect from different number of tethers, *v*_*x*_ was plotted versus *D*^–1^ as shown in Figure 2a for POPC vesicles. It is evident from this graph that the data cluster into groups with similar *D*^–1^ values, and that *v*_*x*_ increases with increasing *D*^–1^. Furthermore, two clusters, characterized by high diffusivity values, are clearly separated from each other, while vesicles with lower diffusivity are more homogeneously distributed. Such distribution of the data is expected from eq 2, since a subpopulation corresponding to a certain number of tethers becomes separated from other subpopulations when the relative change in diffusion is larger than the statistical spread of the diffusion estimation, which is set by the track length *N*_tr_ according to . To achieve this distinction for one, two, and more tethers, a minimum track length of 100 frames was used (Supporting Information, Section 1.4).

**Figure 2:**
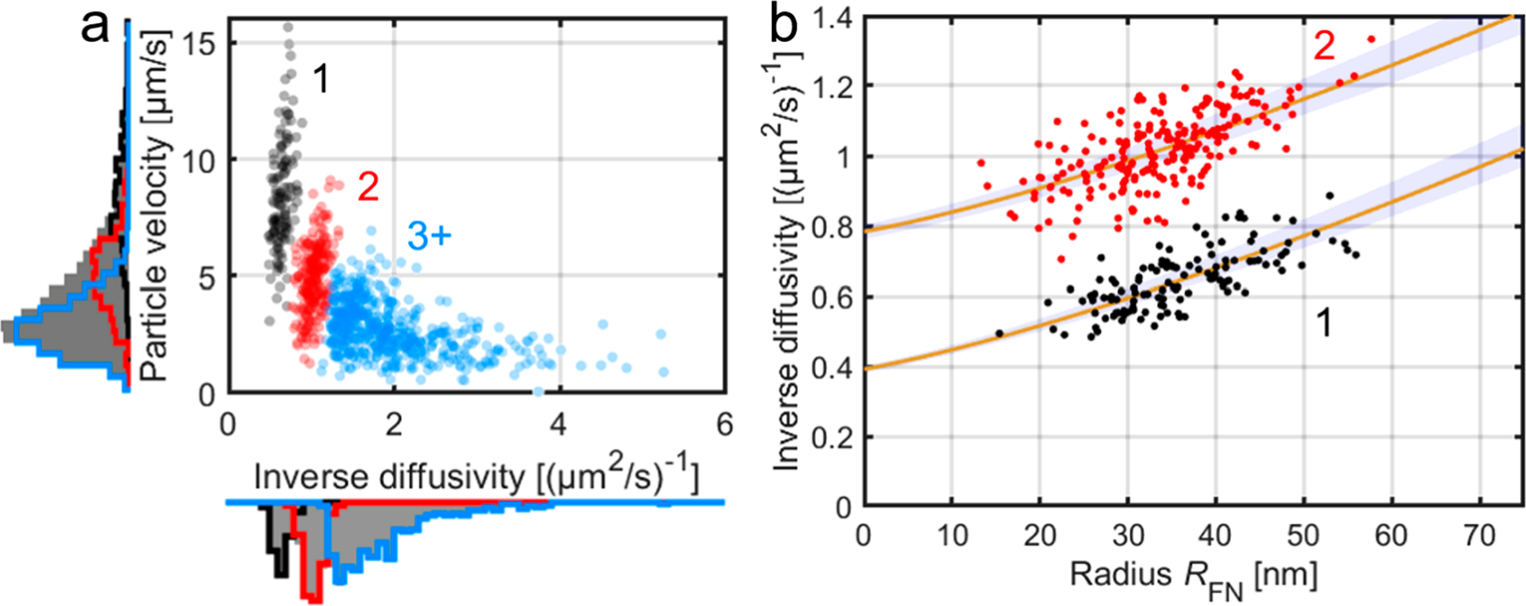
(a) Flow-directional velocity versus inverse diffusivity for POPC vesicles in TE buffer containing 150 mM NaCl. The three different colors designate the selection based on the number of tethers (1, 2, and ≥3), with the solid lines in the histograms representing the contribution from each subpopulation and the gray areas representing the ensemble histograms. (b) Inverse diffusivity versus *R*_FN_ for the first two clusters in (a). The estimated values from the least-squares fit (yellow lines) are *b*_ef_ = 24.8 ± 7.5 nm and *D*_T_ = 2.50 ± 0.07 μm^2^/s (mean ± 95% CI, visualized using the shaded blue region).

Since the radius *R*_FN_ of each vesicle can be determined using eq 1, also the dependence of the measured diffusivity on vesicle size can be inspected. This is in Figure 2b illustrated by plotting *D*^–1^ versus *R*_FN_ for the two clearly separate subpopulations seen in Figure 2a. From this plot, it becomes evident that *D*^–1^ displays a clear dependence on *R*_FN_, with similar slopes for both subpopulations.

Since the observed dependence of *D*^–1^ on *R*_FN_ agrees with the general structure of eq 2, a unique opportunity to compare the measured size dependence with theories for confined nanoparticle diffusion is rendered possible. In general, the mobility of a nanoparticle close to an interface depends on its *R*_h_, the distance from the center of the particle to the interface *h* (Figure 1), and the slip length at the interface *b*_i_, and can be expressed as^1,4,26^
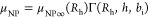
3where μ_NP∞_(*R*_h_) is the mobility of the nanoparticle in bulk and Γ(*R*_h_, *h*, *b*_i_) is the correction factor due to the interface. With the no-slip boundary condition for both interfaces, an explicit expression of the correction factor is given by^1,27^
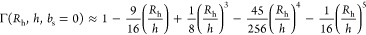
4Since the geometrical size of the particle *R* is the same as *R*_h_ in the case of no slip, the two sizes are often used interchangeably. In our context, the use of *R*_h_ is preferable, because it can naturally be kept when introducing the correction factor representing partial slip.^26^ Furthermore, although Γ is derived under the assumption that *h* ≫ *R*_h_,^1,4^ it is essentially identical to other expressions derived to represent confinement effects in the close proximity regime when *R*_h_/*h* < 0.8 (Supporting Information, Section 2.5).^28^

In the partial-slip case, the hydrodynamics can be described by shifting the no-slip boundary below the interface. Practically, *h* in eq 4 is replaced by the effective height *h*_ef_ = *h* + *b*_i_,^4,14^ whereas the slip at the particle *b*_p_ is implicitly included via . In our case, we can use the small slip-length approximation, *R* ≈ *R*_h_ + *b*_p_, and accordingly, we have *h* ≈ *R*_h_ + *b*_p_ + δ, where δ is the distance from the vesicle to the SLB. Thus, *h*_ef_ = *R*_h_ + *b*_ef_ where *b*_ef_ ≡ δ + *b*_i_ + *b*_p_. With these specifications, the mobility of a particle close to an interface can be expressed as
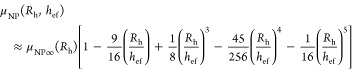
5Considering that *R*_FN_ ≈ *R*_h_ (Supporting Information, Section 2.4), we find that *b*_ef_ can be determined by fitting eq 5 to the 2DFN data.

Taking these specifications into account, a fit using eqs 2 and 5 to the data in Figure 2b yields *b*_ef_ = 24.8 ± 7.5 nm and *D*_T_ = 2.50 ± 0.07 μm^2^/s (mean ± 95% CI), where *D*_T_ is the diffusion coefficient of a single tether. Note that this outcome is valid irrespective of the type of the boundary conditions. More specifically, *b*_ef_ corresponds to the distance δ in the case of no slip, whereas if slip occurs, *b*_ef_ corresponds to the sum of δ and the slip lengths *b*_i_ and *b*_p_. Furthermore, *D*_T_ is in excellent agreement with a diffusion constant of 2.47 ± 0.04 μm^2^/s obtained from fluorescence recovery after photobleaching (FRAP) measurements of fluorescently labeled DNA tethers (Supporting Information, Section 1.5), suggesting that the separate clusters of data points in Figure 2 indeed correspond to subpopulations with one and two tethers. Also note that the mean diffusivity of the single-tether subpopulation is ∼1.4 μm^2^/s, illustrating the significant underestimation one would make if the friction between the vesicle and solution is assumed to be negligible when using nanoparticles in this size regime to quantify the diffusivity of membrane-bound molecules. In addition, since the slope for *D*^–1^ versus *R*_FN_ is similar for both subpopulations (see Figure 2b), the size-dependent friction from the vesicles appears to be identical in the two cases, which in turn suggests that the measured nanoparticle contribution to the diffusion coefficient in the low tethering regime can be used to compensate for the nanoparticle contribution when interpreting diffusion in the multivalent case (*N* > 2).

It is in this context worth noting that the derivation of eq 2 relies on the assumption of independent friction forces. Since the presence of a PEG cushion on the SLB is expected to prevent direct vesicle–SLB interaction, this assumption is reasonable in the case of a single tether; however, in the case of multiple DNA–cholesterol tethers, electrostatic and steric interactions between the linkers cannot be excluded. Using the diffusion constant of a single tether obtained from the FRAP analysis and considering the single-tether population only (Figure 3a–d), the slip length becomes the only free fitting parameter. This was used to assess the generality of the obtained value of *b*_ef_, by analyzing measurements performed for (i) the POPC vesicles at different NaCl concentrations in TE buffer (Figure 3e–g) as well as (ii) cell-derived EVs (Figure 3h) with similar size to that of the POPC vesicles, but with a significantly more complex membrane composition. Using the single-tether population only, *b*_ef_ values of ∼21 to ∼27 and ∼30 nm were obtained for POPC vesicles and EVs, respectively.

**Figure 3:**
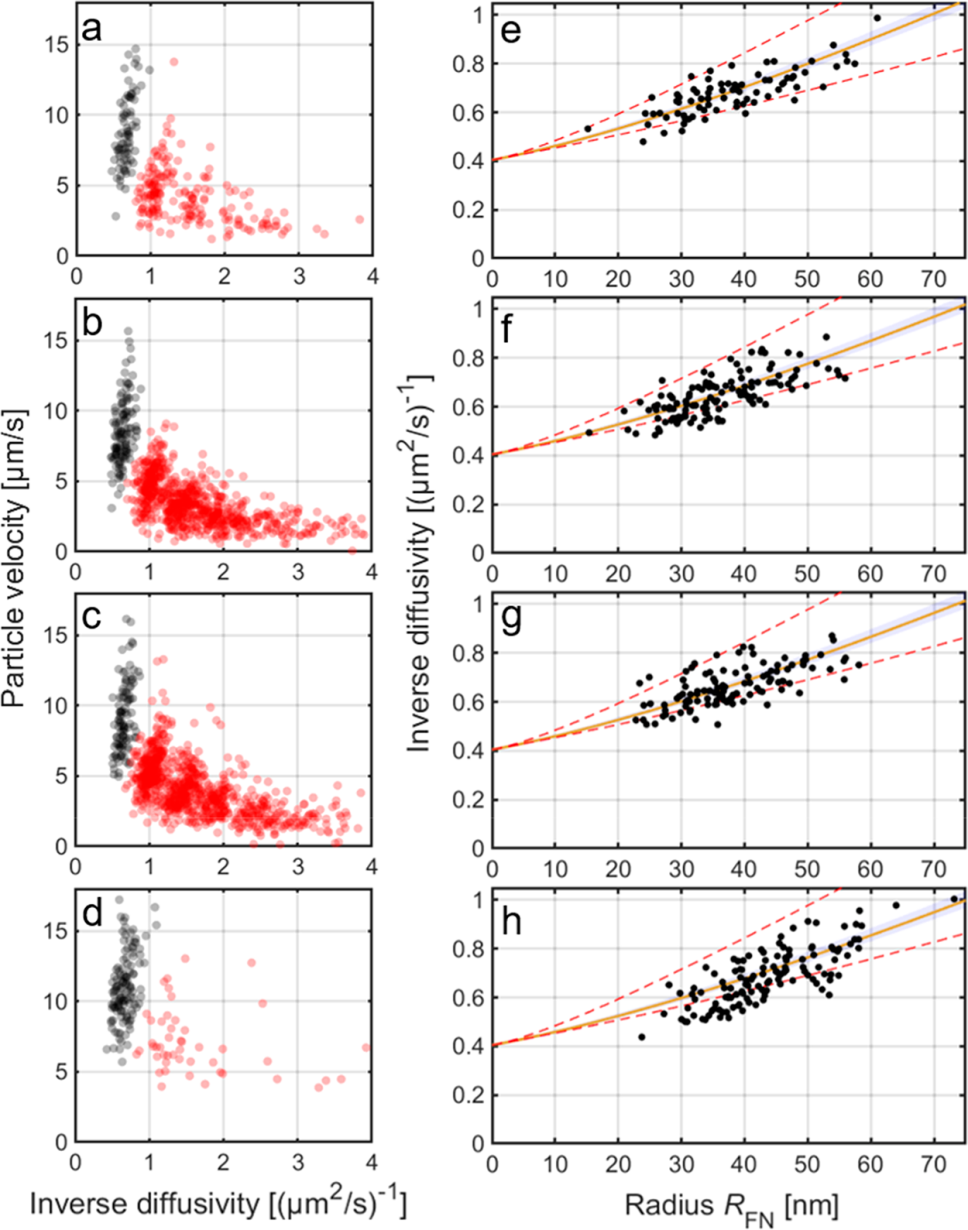
Velocity versus inverse diffusivity (a–d) for all particles, with the single-tether population highlighted in black as well as inverse diffusivity versus *R*_FN_ for the single-tether population (e–h) of POPC vesicles at different NaCl concentrations in the TE buffer: (a,e) 50 mM, (b,f) 150 mM, (c,g) 200 mM NaCl, and (d,h) extracellular vesicles (EVs) in TE buffer with 125 mM NaCl, all fitted using *D*_T_ = 2.47 μm^2^/s (obtained from independent FRAP data). The estimated values from the least-squares fit (yellow lines) are *b*_ef_ = 20.5 ± 3.7, 26.4 ± 5.1, and 27.4 ± 5.3 nm for 50, 150, and 200 mM, respectively, and *b*_ef_ = 30.4 ± 5.8 nm for the EVs (mean ± 95% CI, visualized using the shaded blue region). The red dashed lines correspond to *b*_ef_ = 4 nm (i.e., no slip with δ = 4 nm) and *b*_ef_ = 100 nm. The EV data were adopted from ref (25).

To relate the value of *b*_ef_ (≡δ + *b*_i_ + *b*_p_) to the slip lengths *b*_i_ and *b*_p_, the distance δ needs to be specified. In previous work using 2DFN under similar experimental conditions, it was shown that the nanoparticle size determination was not influenced by neither the length of the linkers nor the flow rate.^21^ This suggests that nanoparticles tethered with flexible linkers are pushed by the flow toward the SLB to a minimum possible distance. To avoid nonspecific interactions between the SLB and the vesicles, we incorporated 0.5 mol% PEG2000-PE within the bilayer for all measurements, which thus defines the distance between vesicles and upper surface of the SLB to the ∼4 nm thickness of this protruding PEG layer.^29^ Under the assumption that the slip length is identical at the POPC SLB and POPC vesicles (*b*_i_ = *b*_p_ = *b̃*), this suggests an effective slip length *b̃* of around 8–12 nm. If the distance between the particles and the surface would not be defined by the PEG cushion but rather by the maximum extension of the cholesterol–DNAlinker (∼15 nm), the analysis would suggest a *b̃* of around 3–6 nm. Further, since the slip lengths for EVs and POPC vesicles are expected to be similar, the most likely reason for the higher *b*_ef_ obtained for EVs is an increase in δ caused by their more complex composition, with protruding proteins and glycans.^7^ This is consistent with the assumption that both POPC vesicles and EVs are indeed pushed by the flow toward the PEG-modified SLB, with molecular protrusions on the EV extending ∼3 to 10 nm from the membrane envelope.

Although being clear from this analysis that the no-slip boundary condition fails to represent the data, with the larger *b*_ef_ for the EVs being in good agreement with expectation of protruding membrane molecules,^7^ it is crucial to recall that a number of assumptions, by necessity, had to be made to perform this analysis. One assumption is the choice of the representation describing the change in diffusivity due to the proximity to a planar interface and in particular the fact that the expression is originally derived under the assumption that the particle is far away from the surface. When comparing *b*_ef_ obtained using different representations of the change in diffusivity, both using a different number of expansion terms for eq 4 as well as the Brenner formula, which is derived to handle the limit of short distances between the particle and the surface,^4,28^ the obtained *b*_ef_ values differ by less than 1.5 nm, which is expected since *R*_h_/*h*_ef_ ≲ 0.75 (Supporting Information, Section 2.5). Another assumption in eq 5 is a neglectable effect from the finite viscosity of the SLB. Confined diffusion is dependent on the viscosity at both sides of the interface, where the magnitude of this effect here is dependent on the ratio between the viscosity of the SLB and surrounding liquid.^30^ However, since the ratio in this case is expected to at least be ∼100,^31^ its contribution to *b*_ef_ is no more than 1 nm (Supporting Information, Section 2.6). A third assumption utilized is that *R*_FN_ = *R*_h_. Since the measured size distribution using 2DFN can be slightly different from the size distribution in bulk, the calibration parameters of eq 1 may introduce an error that affects the obtained *b*_ef_. However, by analyzing the magnitude of this effect using the size distributions measured using NTA, the difference is estimated to be around ∼1 nm for the used vesicle sizes, which in turn contributes to *b̃* by no more than ∼2 nm (Supporting Information, Section 2.4). On the contrary, the approximation *h*_ef_ = *R*_h_ + *b*_ef_, i.e., the linearization of the slip effect, contributes to a slight (≲1–2 nm) underestimation of *b̃* (Supporting Information, Section 2.7).^4^ Combined with the fact that eq 5 is indeed valid for no slip, the obtained *b̃* values are inconsistent with no-slip.

In conclusion, our analysis provides a general approach to experimentally quantify and compare the friction contribution of different nanoparticles when tethered to a lipid membrane. This information makes it possible to clarify the size-dependent mechanistic aspects concerning the mobility of membrane-attached nanoparticles, of importance for systems ranging from viral infections to nanoparticle-assisted drug delivery and mobility quantification of membrane-residing biomolecules using nanoparticle labels. Specifically, we have demonstrated that the size-dependent mobility of SLB-tethered nanoparticles can be quantified using a single measurement if the particle size distribution is sufficiently wide, which is different from previous work that instead relied on measurements of several samples with narrow size distributions.^20^ The possibility to analyze wide size distributions on the individual nanoparticle level without relying on the particle signal–size relation is a key asset for the analysis of biological nanoparticles, such as EVs, exosomes, and viruses, since their size distribution is typically broad, and the fluorescence signal depends strongly on the highly variable membrane composition.^25,32^ The identification of tether subpopulations combined with measurements of both size and diffusivity enabled a direct quantitative comparison with theoretical expressions of the size-dependent mobility. The measurements for POPC and EVs were found not to be well-described by the equations obtained using the conventional no-slip boundary condition. Instead, the obtained *b̃* values for POPC vesicles were similar to the reported slip-length value of 6 ± 0.5 nm for mobile DOPC SLBs,^11^ albeit the latter was obtained for macroscopic interfaces, whereas the slightly larger *b̃* for EVs is likely due to protruding proteins and glycans.^7^

We foresee that the possibility to conduct measurements of this type for not only lipid vesicles but for SLB-tethered nanoparticles in general will inspire the development of refined theoretical approaches for more accurate descriptions of nanoparticle diffusivity in close proximity to mobile interfaces with partial slip, to thereby further the understanding of diffusion of nanoparticles near lipid bilayers.

## Supporting Information Available

Materials and methods, data analysis, and a theoretical description of the mobility of nanoparticles tethered to a planar lipid-bilayer–liquid interface including evaluations of potential bias and uncertainty in the parameter estimation (PDF)
