# Supplementary material for: Diffusion of Lipid Nanovesicles Bound to a Lipid Membrane Is Associated with the Partial-Slip Boundary Condition
Source: Nano Lett. 2021 Aug 17;21(19):8503–9. doi: 10.1021/acs.nanolett.1c02092 (PMC8517973; doi:10.1021/acs.nanolett.1c02092)
Supplement: Supplementary file 1 — nl1c02092_si_001.pdf [file nl1c02092_si_001.pdf]

Supporting Information for

Diffusion of Lipid Nanovesicles Bound to a Lipid Membrane is Associated with the Partial-Slip Boundary Condition

Erik Olsén[1,†,*], Silver Jõemetsa[*,†], Adrián González[1], Paul Joyce[1,2,]

Vladimir P. Zhdanov[1,3], Daniel Midtvedt[4] and Fredrik Höök[1,*]

[1]*Department of Physics, Chalmers University of Technology, Göteborg, Sweden*

[2]*UniSA: Clinical and Health Sciences, University of South Australia, Adelaide, Australia*

[3]*Boreskov Institute of Catalysis, Russian Academy of Sciences, Novosibirsk, Russia*

[4]*Department of Physics, University of Gothenburg, Göteborg, Sweden*

[†]) These authors contributed equally

[*] Corresponding authors: olsene@chalmers.se, fredrik.hook@chalmers.se

1 Materials and methods

1.1 Preparation of lipid vesicles

1-palmitoyl-2-oleoyl-sn-glycero-3-phosphocholine (POPC), PEG2000-PE: 1,2-dioleoyl-sn-glycero-3-phosphoethanolamine-N-[methoxy(polyethylene glycol)-2000] (PEG), and the fluorescent head-group labeled 1,2-dioleoylsn-glycero-3-phosphoethanolamine-N-(lissamine rhodamine B sulfonyl) (Rh-PE) lipids were provided by Avanti Polar Lipids Inc., whereas the fluorescently head-group labeled lipids 1,2-Dipalmitoyl-sn-glycero-3-phosphoethanolamine labeled with Atto 488 (ATTO488PE) and 1,2-Dipalmitoyl-sn-glycero-3-phosphoethanolamine labeled with Atto 647N (ATTO647PE) were both provided by ATTO-TEC GmbH. The DNA tethers were formed using 4 cholesterol-terminated single-stranded DNA obtained from Eurogentec S.A. (Seraing, Belgium). These strands are complementary to each other in such a way that they self-assemble into a 39 bp double-stranded DNA with two cholesterol molecules at each end. The sequences of the single strands are as follows: 5'-T*GG-ACA-TCA-GAA-ATA-AGG-CAC-GAC-GGA-CCC-3' with cholesteryl-TEG in 3'-end we will refer to this strand as ssA; 5'-CCC-TCC-GTC-GTG-CCT-3' with cholesteryl-TEG in 5'-end (ssB); 5'-TAT-TTC-TGA-TGT-CCA-AGC-CAC-GAG-TTC-CCC-3' (ssC); 5'-CCC-GAA-CTC-GTG-GCT-3' with cholesteryl-TEG in 5'-end (ssD).

The 2 mol% ATTO488PE-labeled POPC vesicles were prepared by the freeze–thaw extrusion method. Specifically, the appropriate lipid mixture was dissolved in chloroform, the mixture was then evaporated at the bottom of a round flask under vacuum for >2 h to remove the solvent. After vacuum drying, the lipid mixture was rehydrated in a TE buffer (50 mM NaCl (Sigma Aldrich), 10 mM TRIS (Merck), 1 mM Na_2EDTA (Sigma Aldrich), adjusted to pH = 7.4 using HCl) to a concentration of 1 mg/mL and vortex mixed to dissolve the lipids. The vesicle solution was

thereafter freeze-thawed 5 times and extruded through a 30 nm polycarbonate membrane (Whatman) 31 times using an Avanti mini extruder. Vesicles for supported lipid bilayer (SLB) formation (see Section 1.3) were extruded through a 50 nm polycarbonate membrane (Whatman) 11 times using the mini extruder. Lipid solutions were then stored at 4 °C until use.

The data of the extracellular vesicles (EVs) were taken from Ref. [1], in which the relation between their fluorescent intensity and size was inspected. For further details regarding the EV preparation, see Ref. [1].

1.2 Microfluidic channel fabrication

Borosilicate cover glasses (Menzel-Gläser, D263®, #1.5) were prepared using detergent-assisted cleaning, by treating the glass in a 10% 7X cleaning solution (MP Biomedicals) at 85 °C for at least 1 h, followed by rinsing with MilliQ water and drying with nitrogen. The microfluidic channels (80 µm high, 400 µm wide and 14 mm long) were prepared from a PDMS [Poly(dimethylsiloxane), Sigma-Aldrich] pre-polymer by mixing the elastomer and the curing agent at a mass ratio of 10:1 and degassing for 15 min in a desiccator. After pouring the degassed PDMS mixture on the master mold (defining the channel geometry), it was subsequently baked at 85 °C for 2 hours. The PDMS was removed from the mold and 0.5 mm holes were punched at the ends of each channel for subsequent tubing connection. These punched slabs were then bonded to the cover glass using O_2 plasma for 45 s (0.2 mBar, 30 SCCM, 100 W, Atto Plasma Chamber, Diener Electronic GmbH, Germany) with a subsequent baking step at 80 °C for 5 min. Steel pins were inserted in the holes as adapters for Teflon tubing, where pieces of Tygon tubing were used to interconnect the aforementioned.

1.3 Supported lipid bilayer formation and tethering

The SLB (consisting of the following vesicle mixtures: (i) 98 mol% POPC and 2 mol% Rh-PE; (ii) 0.5 mol% PEG and 99.5 mol% POPC, mixed together with a ratio of 1:1000 to a lipid concentration of 0.2 mg/mL) was formed on the floor of a microfluidic channel followed by incubation with a solution of 200 nM cholesterol-DNA (made by pre-hybridization of the ssA and ssB strands) for at least 15 min in a 125 mM NaCl TE buffer environment.

Vesicles were tethered to the SLB using cholesterol-modified DNA strands as described previously.[2] Specifically, vesicles were incubated with cholesterol-DNA (made by pre-hybridization of the ssC and ssD strands in a 50 mM NaCl TE buffer) at a ratio of approximately 3 DNA/vesicle with lipid concentration being 0.25 mg/ml. The mixture of vesicles and DNA was subsequently diluted to 0.0025 mg/ml (100x) and injected into the PDMS microfluidic channel in a 50 mM NaCl TE buffer, and the sequency specific DNA-hybridization controlled tethering was monitored in real time using total internal reflection fluorescence (TIRF) microscopy.

Once a sufficient surface coverage was obtained, the vesicle tethering was terminated by injecting TRIS buffer, followed by two-dimensional flow nanometry (2DFN) measurements (see below) in TE buffer containing either 50 mM, 150 mM or 200 mM NaCl. For the 2DFN measurements using EVs, the TE buffer contained 125 mM NaCl. All 2DFN measurements were performed at a 30 μL/min flow rate.

1.4 Fluorescence imaging and 2DFN data analysis

TIRF microscopy experiments were conducted using an inverted Eclipse Ti-E microscope (Nikon Corporation), equipped with a Perfect Focus System (PFS), a CFI Apo TIRF 100x oil objective (NA 1.49), a Lumencor Spectra X LED light source and an Andor iXon EM+ DU-897 EMCCD

camera. A TRITC filter set (Semrock) was used for visualizing the SLB (containing Rh-PE), a FITC filter set (Semrock) was used to visualize the ATTO488 labeled vesicles, and a Cy5 filter set (Chroma Technology) was used to visualize ATTO647 labeled vesicles. The acquisition rates were over 30 f.p.s at all times.

All data processing was performed using MATLAB (MathWorks), with the particle tracking analysis being performed using custom-written analysis software as previously described in Ref.[3], where the details for how the diffusion constant was calculated from the mean squared displacement are outlined in Section 2.1. The minimum particle track length was 100 frames, and only particles for which the diffusion constant estimate in the flow direction was less than 20% apart from the diffusion constant estimate in the orthogonal direction were included. The second threshold was used to exclude vesicles which interact with potential defects in the SLB or change the number of tethers with the SLB during the trace.[4]

The clusters corresponding to one, two and more tethers were first identified visually using the inverse diffusivity and particle velocity plots (see Figures 2-3 in Main Text). This visual separation was possible due to that the statistical spread of the diffusion estimation, which is set by the track length N_{tr} according to $\Delta D \approx D/\sqrt{N_{\mathrm{tr}}}$, was less than the diffusivity difference between particles with one, two and more tethers due to the minimum track length of 100 frames.The visual separation was subsequently refined based on a fit of the clustered data using Eq. 2 in the Main text, making possible to refine the cluster corresponding to 2 tethers to include values corresponding to particles with 2±0.5 tethers, for which the obtained result did not noticeably change.

The numerical fits to quantify b_{ef} were performed using the viscosity of water at 22 °C and the relation between drift velocity and inverse diffusivity (see Figure 2(a) in Main Text) instead of size and inverse diffusivity, although the latter are in the Main Text used to display the fits (see Figure 3 in Main Text). This was done because drift velocity and inverse diffusivity are to a good approximation independent values, whereas the size estimation using 2DFN is dependent of the inverse diffusivity (see Eq. 1 in Main Text).

1.5 Fluorescence recovery after photobleaching

Fluorescence recovery after photobleaching (FRAP) was used to determine the diffusivity of the cholesterol-DNA tethers inserted into the SLB in the absence of liposomes. This was done to provide a comparison to the obtained tether diffusion value from 2DFN. The FRAP value of the cholesterol-DNA diffusion was also used for the fits presented in Figure 3 in the Main Text.

For the FRAP experiments an SLB with the same composition as the SLBs of the 2DFN experiments was formed on the same type of silica substrate. The SLB was incubated using the same DNA tether concentration and time as in the 2DFN. The DNA tether used was very similar to the one described in section 1.1, with the difference that ssA was labeled with TAMRA (560/575 nm) as a substitution of the thymine base marked with an asterisk and ssC and ssD lacked cholesterol moieties to avoid the possibility that both ends of the ds-DNA constructs self-insert into the SLB in the absence of vesicles. FRAP diffusivity measurements were performed using the TIRF microscope described in Section 1.4. To create circularly symmetric bleached areas with a spot diameter of ~20 μm, a Kr-Ar mixed gas ion laser (Stabilite 2018, Spectra-Physics Lasers) at a wavelength of 531 nm was used to illuminate such a spot in the field of view for 5 s. Three frames were collected before photobleaching and the recovery after photobleaching was recorded in stagnant conditions for 5 min with a frame interval of 1 s. The acquired images were analyzed

using the software and method reported in Ref. [5] to extract the diffusivity of the fluorophores. The FRAP experiments showed a diffusivity of 2.47±0.04 μm^2/s (the uncertainty represents the standard error of the mean). We ascribe this result to the mobility of a single DNA tether self-inserted into the SLB via the two cholesterol moieties positioned at the ends of ssA and ssB.

2 Estimations of potential bias and uncertainty in the parameter estimation

2.1 Correction between mean squared displacement and diffusion constant

The particle diffusion constant was estimated using the mean squared displacement. In this case, both localization error and exposure time affect the mean squared displacement as described in Ref. [6]:

$$< (\Delta x_n)^2 > = 2D\Delta t + 2(\sigma^2 - 2DK\Delta t), \quad \text{(S1)}$$

$$< \Delta x_n \Delta x_{n+1} > = -(\sigma^2 - 2DK\Delta t), \quad \text{(S2)}$$

$$< \Delta x_n \Delta x_m > = 0 \text{ for } |m - n| > 1, \quad \text{(S3)}$$

where Δx_n is the particle displacement between frames, σ is the localization error and K is a factor describing the position uncertainty originating from the exposure time.[6] If the exposure time is set by the time between frames, then $K = 1/6$.[6]

Although it is possible to quantify the correction term for each tracked particle, $< \Delta x_n \Delta x_{n+1} >$ is a statistical estimate which uncertainty depends on the particle track length.[6] Thus, the bias contribution $< \Delta x_n \Delta x_{n+1} >$ was here analyzed as a function of $< (\Delta x_n)^2 >$ using a linear ensemble fit for all the analyzed particles (see Figure S1). The uncertainty in the correction term results in a minor variation in estimated b_{ef} (within ± 2 nm) as it slightly affects the diffusion quantification of the tracked particles.

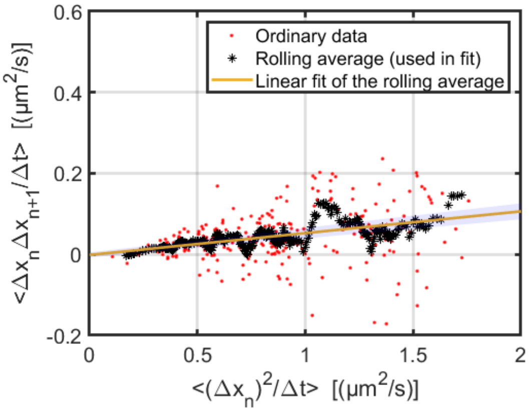

Figure S1: Quantification of bias contribution when estimating the diffusion constant from the mean squared displacement. A least-squares fit using a linear function (y=Ax+C) and a 10 data point rolling average (yellow line) gives A=0.0538 ± 0.0067 and C= -0.0013 ± 0.0063 (mean ± 95% CI, visualized using the shaded blue region).

2.2 Assumption of neglectable deformations of the SLB-tethered vesicles

In the 2DFN analysis, the particles are assumed to be spherical. Since the particles are not solid, they can potentially deform both due to the flow-induced force as well as the attachment to the SLB. However, it has been previously reported that when using positively charged vesicles with a size of ~45 nm attached to a negatively charged SLB, negligible deformation of the vesicles was observed.[7] In the case of one or two tethers between the vesicles and the SLB, the associated force is expected to be significantly weaker than in the case of differently charged lipid interfaces. Thus, such source of deformation is negligible.

In the case of flow-induced deformation, the scale of the related force to deform a vesicle is

$$F_{\mathrm{def}} = \frac{E_b \Delta h}{R^2} = \frac{8\pi\kappa\Delta h}{R^2}, \qquad \text{(S4)}$$

where R is its initial radius and Δh is the reduced height from the deformation. Using $\kappa \approx 25 k_B T$,[8] $\Delta h/R = 0.02$ and $R = 50$ nm, $F_{def} \approx 1$ pN. Compared to the flow-induced force (see Figure S2), F_{def} is more than one order of magnitude larger, thus flow-induced deformation is also negligible. In fact, κ can be much larger than $25 k_B T$,[8] and accordingly the ratio of F_{def} to the flow-induced force can be much larger, and the effect of flow-induced force on the vesicle shape can be even smaller.

2.3 Determination of calibration parameters and its effect for the b_{ef} estimation

In Eq. 1 in the Main Text there are two calibration parameters, λ and A. From previous work, the value of λ was determined to be 24.4 nm.[3] To determine A, the calibration was performed by associating the maximum of the distribution of the hydrodynamic radius, R_h, obtained from nanoparticle tracking analysis (NTA) of suspended nanoparticles (Nanosight LM10 HS, Malvern), with the maximum of the measured $v_x \mu^{-1}$ distribution, enabling A to be determined from a fit as illustrated in Figure S2.

In previous work, λ in Eq. 1 was attributed to the sum of partial slip and the distance between the particle and the interface of the SLB.[3] It is therefore instructive to evaluate how the value of λ influences the determination of b_{ef} made in the Main Text. As shown in Figure S2, when λ is varied the size-force fit remains similar, this since the value of A changes during the fit to compensate the difference in λ. If λ is varied approximately ±10 nm, b_{ef} only varies by ± 2.5 nm following the data analysis approach outlined in the Main Text, with increasing λ resulting in lower b_{ef} values. Thus, the value of λ does not influence the value of b_{ef} in the subsequent analysis.

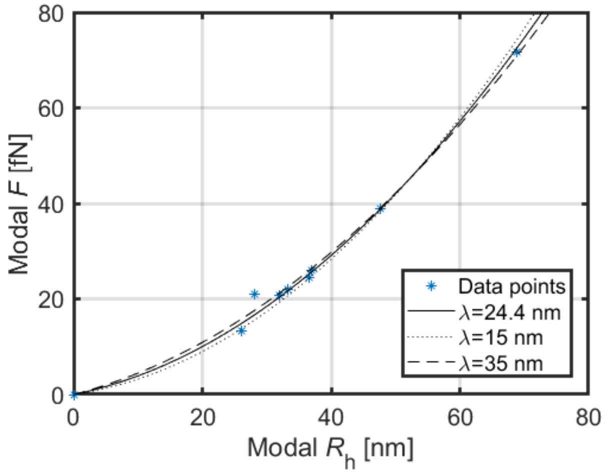

Figure S2: The effect of different λ parameters (approximately ±10 nm) during the calibration step of Eq. 1 in the Main Text, where the modal hydrodynamic radius measured in bulk using NTA is related to the measured modal force measured using 2DFN. For these different λ parameters, the fitted b_{ef} for POPC vesicles in 150 mM NaCl containing TE buffer (see Figure 3(b) and (f) in Main Text) is 26.4 nm (λ =24.4 nm), 28.9 nm (λ =15 nm) and 24.5 nm (λ =35 nm). A full least-squares fit for both A and λ results in a λ of 29.7 nm.

2.4 Difference in size determination using 2DFN and bulk NTA

Since 2DFN relies on the flow-induced force of particles bound to an SLB to estimate the particle size, R_{FN} is not strictly the same as R_{h}. One of the reasons to this difference is that the attachment rate of nanoparticles to an SLB is controlled by diffusion. Specifically, the particle diffusion flux to the surface is proportional to ${R_{\mathrm{h}}}^{-\beta}$, where β is 1/2 in the no-flow case and 2/3 in the case of high flow.[9] In our case, the particles are deposited during flow. Thus, $\beta = 2/3$ and the vesicle size distribution at the surface, $\phi(R_{\mathrm{h}})$, is proportional to $\rho(R_{\mathrm{h}})/{R_{\mathrm{h}}}^{2/3}$, where $\rho(R_{\mathrm{h}})$ is the R_{h} distribution in bulk.

A second contribution to this difference between R_{FN} and R_{h} is that not all particles on the surface are measured during a 2DFN measurement. Since a 2DFN measurement consists of a video

containing ~10^4 frames of a fixed imaging region, with the video length being considerably longer than the average particle track length, the measured particle size distribution, $\Gamma(R_\mathrm{h})$, is skewed towards particles with higher flow-induced velocities. This means, in turn, that $\Gamma(R_\mathrm{h}) \propto \phi(R_\mathrm{h})v(R_\mathrm{h})$. During the calibration measurements, the force distribution refers to all analyzed particles, and not those belonging to a particular tether population. According to Eq. 1 in the Main Text, $v \propto FD$, where D depends on both μ_NP, μ_T and N, and $< N >$ depends on particle size as larger vesicles, on average, are expected to contain more tethers. The calibration measurements (Figure S2) represent vesicles incubated with cholesterol-DNA at a ratio of approximately 3-6 DNA/vesicle, with increasing average number of tethers with increasing particle size. As seen in Figure S3(a-b), D values scale differently with R_FN for different vesicle sizes, which is attributed to the relation between average number of tethers and vesicle size. For the larger vesicles containing ~6 DNA tethers, $D \approx 1/R_\mathrm{FN}$, whereas for smaller vesicles containing approximately ~3 DNA tethers, $D \approx 1/R_\mathrm{FN}^{0.2}$. Since the calibration consists of vesicles with an average number of tethers within the range shown in Figure S3(a-b), the effective scaling between D and R_FN is between the two extremes. Thus, to get an upper estimate of the difference between R_FN and R_h, we approximate that $v \propto D \approx 1/R_\mathrm{h}$ (the effect of a weaker size dependence seen in Figure S3(a) is discussed below). This suggests that $\Gamma(R_\mathrm{h})$ is proportional to $\rho(R_\mathrm{h})F(R_\mathrm{h})/{R_\mathrm{h}}^{5/3}$.

Finally, during the calibration procedure, the histogram of the hydrodynamic force is binned as a function of force and compared to NTA which is binned according to hydrodynamic radius. This means that in the calibration, $\rho(R_\mathrm{h})$ is compared with $f(F) \propto \Gamma\big(R_\mathrm{h}(F)\big)\frac{\mathrm{d}R_\mathrm{h}(F)}{\mathrm{d}F}$, where $\frac{\mathrm{d}R_\mathrm{h}(F)}{\mathrm{d}F}$ is the Jacobian for the change in sampling variable. Thus, taking these experimental aspects into account, the hydrodynamic particle size corresponding to the modal force is determined by the maximum of the following function:

$$g(R_\mathrm{h}) \propto \frac{\rho(R_\mathrm{h})}{{R_\mathrm{h}}^{5/3}} \frac{F(R_\mathrm{h})}{\frac{\mathrm{d}F(R_\mathrm{h})}{\mathrm{d}R_\mathrm{h}}}. \qquad \text{(S5)}$$

If $F(R)$ would be the same in Eq. 1 in the Main Text, then $g(R_\mathrm{h}) \propto \frac{\rho(R_\mathrm{h})}{{R_\mathrm{h}}^{2/3}} \frac{R_\mathrm{h}+\lambda}{2R_\mathrm{h}+\lambda}$. To quantify the potential difference in size determination using NTA and 2DFN, $\rho(R_\mathrm{h})$ was estimated using log-normal deconvolution for NTA data of the POPC vesicles used in the Main Text,[10] with both $\rho(R_\mathrm{h})$ and $g(R_\mathrm{h}) \propto \frac{\rho(R_\mathrm{h})}{{R_\mathrm{h}}^{2/3}} \frac{R_\mathrm{h}+\lambda}{2R_\mathrm{h}+\lambda}$ being presented in Figure S3(c-d). In this example, the maximum of $g(R_\mathrm{h})$ occurs for vesicles that are ~1.7 nm smaller than the maximum of $\rho(R_\mathrm{h})$.

If D would instead have a weaker size dependence than the assumed $D(R_\mathrm{h}) \propto 1/R_\mathrm{h}$, (c.f. Figure S3(a)), the difference in maxima positions between $\rho(R_\mathrm{h})$ and $g(R_\mathrm{h})$ is than the example shown in Figure S3(c-d). In the case of $v \propto D \approx 1/R_\mathrm{h}^{0.2}$, the difference in maxima positions between $\rho(R_\mathrm{h})$ and $g(R_\mathrm{h})$ becomes instead ~0.2 nm.

The same trend holds true if β would be less than 2/3. For example, if $\beta = 1/2$ while $D(R_\mathrm{h}) \propto 1/R_\mathrm{h}$ the difference in maxima positions would be ~1.4 nm. Thus, the value of ~1.7 nm is an upper estimate for ~30 nm radius vesicles, particularly considering that the calibration measurements around ~30 nm radius use 3 DNA/vesicle. Therefore, the difference is most likely between the two extremes, i.e. around 1 nm.

If one assumes that the maxima of NTA and 2DFN occurs for the same particle size, which is the case during the calibration step, then this analysis suggests that the measured R_FN represents a slight overestimation of R_h. In the context of slip-length estimation, if the size is overestimated by ~1 nm, its contribution to $\tilde{b}$ is approximately 2 nm.

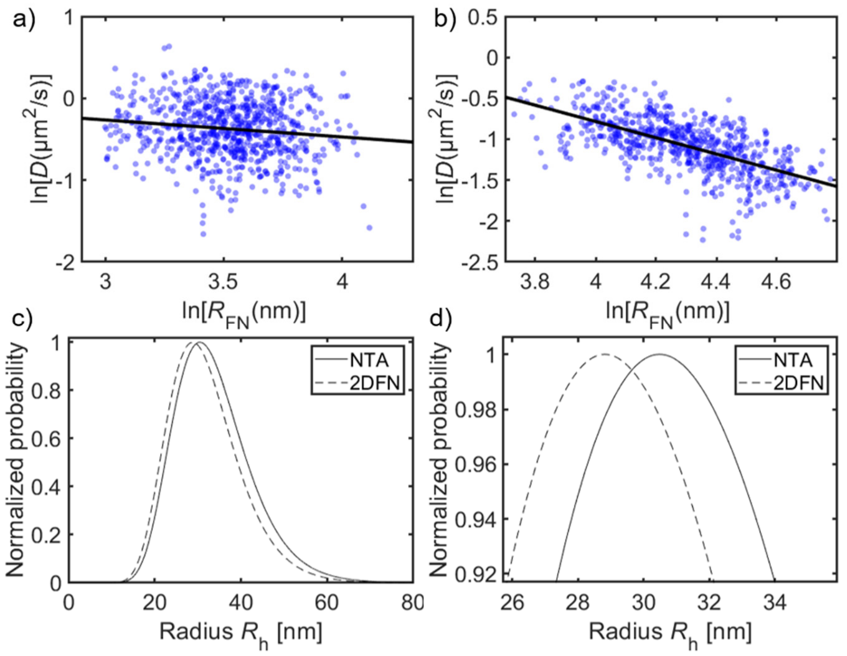

Figure S3: Estimation of how the average particle diffusivity scales with size and the estimated difference in size estimation between NTA and 2DFN. (a-b) Two different 2DFN measurements of POPC vesicles with different sizes and different number of tethers between the particle and SLB, where (a) on average contain 3 tethers with a mode R_{FN} of ~30 nm and (b) on average contain 6 tethers with a mode R_{FN} of ~70 nm. The log-log plot (a) results in a slope of -0.21 ± 0.11 and (b) results in a slope of -1.00 ± 0.09. (c-d) Track-length-corrected size distribution from NTA $\rho(R_{\mathrm{h}})$ and the corresponding estimated force distribution $g(R_{\mathrm{h}}) \propto \frac{\rho(R_{\mathrm{h}})}{R_{\mathrm{h}}^{2/3}} \frac{R_{\mathrm{h}}+\lambda}{2R_{\mathrm{h}}+\lambda}$. (c) contains the full distributions whereas (d) is the same as (c) with the peaks magnified. The maximum of $g(R_{\mathrm{h}})$ occurs for particles ~1.7 nm smaller than the maximum of $\rho(R_{\mathrm{h}})$.

2.5 Effect of using different expressions for confined diffusion

To evaluate if the choice of expression for the confined diffusion affects the obtained b_{ef} (while assuming the small slip approximation; see Main Text), both the first and fifth order of the Faxén expansion and the Brenner formula[11] were fitted to the data and presented in Figure S4. The choice of model has insignificant influence on the observed relation, i.e., within ± 1.5 nm, which is

expected since $R_h/h_{ef} \lesssim 0.75$, $h_{ef} = R_h + b_{ef}$, where for such small R_h/h_{ef} the different expressions for the confined diffusion are similar.[12]

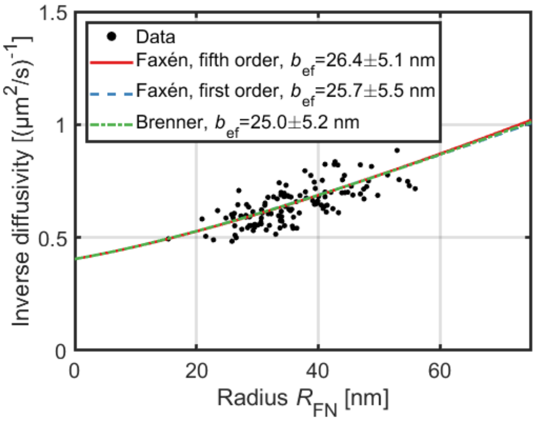

Figure S4: Fits to quantify b_{ef} using different expressions for the confined particle diffusion (both first and fifth order of the Faxén approximation and the Brenner formula[11]). The b_{ef} values from the different expressions are within ± 1 nm. Data: POPC vesicles in 150 mM NaCl TE buffer (see Figure 3(b) and (f) in Main Text).

2.6 Effect on confined diffusion from a slightly mobile interface

In Eq.5 in the Main Text, the expression for confined particle diffusion is based on the assumption that the planar interface is immobile. However, since the lipids in the bilayer can diffuse around, the viscosity in the bilayer is finite. According to Ref.[13], when the surface has a finite viscosity but a no-slip boundary condition, the confinement correction factor is expressed in terms of

$$\frac{R}{h}\frac{\frac{2}{3}-\eta_2/\eta_1}{1+\eta_2/\eta_1}, \qquad \text{(S6)}$$

where η_1 is the viscosity of the medium in which the particle is located in, η_2 is the viscosity of the second medium (at the other side of the interface) and h is the distance from the center of the

particle to the interface. In the case of lipid membranes, the viscosity ratio between lipid membranes and the surrounding water is approximately $\eta_2/\eta_1 \approx 100$,[14] which results in

$$\frac{R}{h}\frac{\frac{2}{3}-\eta_2/\eta_1}{1+\eta_2/\eta_1} \approx \frac{-R}{1.02h}. \quad \text{(S7)}$$

The sizes used in the experiments presented in the Main Text are such that h is around 20-60 nm, thus a factor of 1.02 contributes about 1 nm to b_{ef}.

2.7 Effect of small-slip approximation

The small slip approximation is the same as linearizing the effect of the slip length. The full expression describing confined diffusion with partial slip at a planar interface deviates from the linear behavior at large slip lengths.[15] To estimate the effect of using the small slip approximation in the case of $b_{\mathrm{p}} = 0$, both the full expression and the linearized approximation are plotted as a function of Knudsen number, b/h, in Figure S5. Since the linearized curve is always lower than the full expression, the linearization overestimates the effect of the slip on the resulting diffusion. Similarly, the approximation $R_{\mathrm{h}} \approx R - b_{\mathrm{p}}$ also overestimates the effect of the slip on the resulting nanoparticle diffusion. This in turn infers that linearizing the slip contributes to a slight underestimation of the actual slip length.

To estimate the magnitude of this underestimation, we first assumed that $b = 5$ nm was obtained using the linearized model for the confined diffusion, with $h = 50$ nm. To obtain the same I value using the full model with the same h, $b \approx 6.0$ nm. Similarly, if $R_{\mathrm{h}} = 40$ nm and $b = 5$, one obtains $R = 45$ nm using the linearized expression. To obtain the same R value using the full expression

with the same $R_\mathrm{h} = 40$, one needs $b \approx 6.7$ nm. Thus, the linearization underestimates $\tilde{b}$ by approximately 1-2 nm.

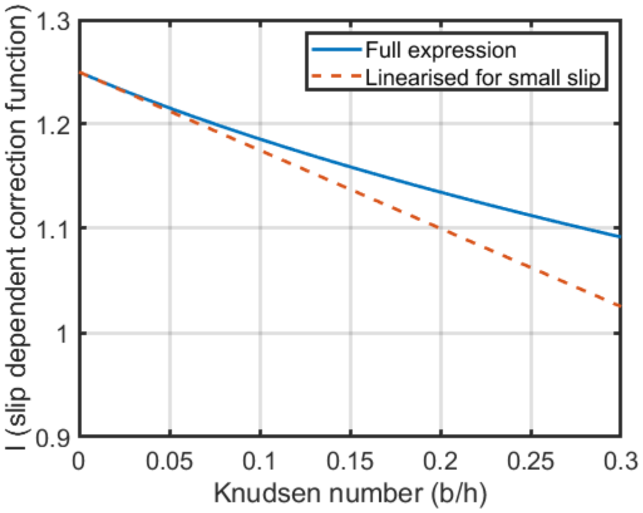

Figure S5: Plot of both the small slip model and the full slip model in Ref.[15] as a function of Knudsen number (b/h). Since the linearized curve is always lower than the full expression, the linearization contributes to an underestimation of the slip length.

References

(1) Jõemetsa, S.; Joyce, P.; Lubart, Q.; Mapar, M.; Celauro, E.; Agnarsson, B.; Block, S.; Bally, M.; Esbjörner, E. K.; Jeffries, G. D. M.; Höök, F. Independent Size and Fluorescence Emission Determination of Individual Biological Nanoparticles Reveals That Lipophilic Dye Incorporation Does Not Scale with Particle Size. *Langmuir* **2020**, *36* (33), 9693–9700. https://doi.org/10.1021/acs.langmuir.0c00941.

(2) Simonsson, L.; Jönsson, P.; Stengel, G.; Höök, F. Site-Specific DNA-Controlled Fusion of Single Lipid Vesicles to Supported Lipid Bilayers. *ChemPhysChem* **2010**, *11* (5), 1011–1017. https://doi.org/10.1002/cphc.200901010.

(3) Block, S.; Fast, B. J.; Lundgren, A.; Zhdanov, V. P.; Höök, F. Two-Dimensional Flow Nanometry of Biological Nanoparticles for Accurate Determination of Their Size and Emission Intensity. *Nat. Commun.* **2016**, *7* (1), 12956. https://doi.org/10.1038/ncomms12956.

(4) Block, S.; Zhdanov, V. P.; Höök, F. Quantification of Multivalent Interactions by Tracking Single Biological Nanoparticle Mobility on a Lipid Membrane. *Nano Lett.* **2016**, *16* (7), 4382–4390. https://doi.org/10.1021/acs.nanolett.6b01511.

(5) Jönsson, P.; Jonsson, M. P.; Tegenfeldt, J. O.; Höök, F. A Method Improving the Accuracy of Fluorescence Recovery after Photobleaching Analysis. *Biophys. J.* **2008**, *95* (11), 5334–5348. https://doi.org/10.1529/biophysj.108.134874.

(6) Vestergaard, C. L.; Blainey, P. C.; Flyvbjerg, H. Optimal Estimation of Diffusion Coefficients from Single-Particle Trajectories. *Phys. Rev. E* **2014**, *89* (2), 022726. https://doi.org/10.1103/PhysRevE.89.022726.

(7) Tabaei, S. R.; Gillissen, J. J. J.; Block, S.; Höök, F.; Cho, N. J. Hydrodynamic Propulsion of Liposomes Electrostatically Attracted to a Lipid Membrane Reveals Size-Dependent Conformational Changes. *ACS Nano* **2016**, *10* (9), 8812–8820. https://doi.org/10.1021/acsnano.6b04572.

(8) Jackman, J. A.; Avsar, S. Y.; Ferhan, A. R.; Li, D.; Park, J. H.; Zhdanov, V. P.; Cho, N. J. Quantitative Profiling of Nanoscale Liposome Deformation by a Localized Surface Plasmon Resonance Sensor. *Anal. Chem.* **2017**, *89* (2), 1102–1109. https://doi.org/10.1021/acs.analchem.6b02532.

(9) Olsson, T.; Zhdanov, V. P.; Höök, F. Total Internal Reflection Fluorescence Microscopy for Determination of Size of Individual Immobilized Vesicles: Theory and Experiment. *J. Appl. Phys.* **2015**, *118* (6). https://doi.org/10.1063/1.4928083.

(10) Saveyn, H.; De Baets, B.; Thas, O.; Hole, P.; Smith, J.; Van der Meeren, P. Accurate Particle Size Distribution Determination by Nanoparticle Tracking Analysis Based on 2-D Brownian Dynamics Simulation. *J. Colloid Interface Sci.* **2010**, *352* (2), 593–600. https://doi.org/10.1016/j.jcis.2010.09.006.

(11) Bian, X.; Kim, C.; Karniadakis, G. E. 111 Years of Brownian Motion. *Soft Matter* **2016**, *12* (30), 6331–6346. https://doi.org/10.1039/c6sm01153e.

(12) Goldman, A. J.; Cox, R. G.; Brenner, H. Slow Viscous Motion of a Sphere Parallel to a Plane Wall-I Motion through a Quiescent Fluid. *Chem. Eng. Sci.* **1967**, *22* (4), 637–651. https://doi.org/10.1016/0009-2509(67)80047-2.

(13) Lee, S. H.; Chadwick, R. S.; Leal, L. G. Motion of a Sphere in the Presence of a Plane Interface. Part 1. An Approximate Solution by Generalization of the Method of Lorentz. *J. Fluid Mech.* **1979**, *93* (4), 705–726. https://doi.org/10.1017/S0022112079001981.

(14) Saffman, P. G.; Delbrück, M. Brownian Motion in Biological Membranes. *Proc. Natl. Acad. Sci.* **1975**, *72* (8), 3111 LP – 3113. https://doi.org/10.1073/pnas.72.8.3111.

(15) Lauga, E.; Squires, T. M. Brownian Motion near a Partial-Slip Boundary: A Local Probe of the No-Slip Condition. *Phys. Fluids* **2005**, *17* (10). https://doi.org/10.1063/1.2083748.
